# Association of *ATM, CDH1* and *TP53* genes polymorphisms with familial breast cancer in patients of Khyber Pakhtunkhwa, Pakistan

**DOI:** 10.4314/ahs.v22i3.17

**Published:** 2022-09

**Authors:** Abdur Rahim, Zakiullah, Asif Jan, Johar Ali, Fazli Khuda, Basir Muhammad, Hamayun Khan, Hussain Shah, Rani Akbar

**Affiliations:** 1 Department of Pharmacy University of Peshawar, Pakistan; 2 Department of Pharmacy Abasyn University, Peshawar, Pakistan; 3 Usman Institute of Technology University, Block 7, Gulshan-e-iqbal, Abul Hasan road, Karachi; 4 Atomic Energy Cancer Hospital Swat Institute of Nuclear Medicine, Oncology & Radiotherapy; 5 Department of Pharmacy, Adul Wali Khan University Mardan, Pakistan

**Keywords:** Breast cancer, TP53, CDH1, ATM, Risk Allele, Genotype

## Abstract

**Background:**

Genetic studies play a significant role in understanding the underlying risk factors of breast cancer. Polymorphism in the tumor suppressor gene TP 53, CDH1 and ATM genes are found to increase susceptibility for breast cancer globally.

**Objective:**

This study aimed to identify/analyze the contribution of genetic polymorphisms in the breast cancer candidate genes ATM, TP53 and CDH1 that may be associated with familial breast cancer risk in the Khyber Pakhtunkhwa population.

**Subjects and Methods:**

In the present case-control study, Whole Exome Sequencing (WES) of the 100 breast cancer patients and 100 ethnic controls were performed for the selected genes in the target population.

**Results:**

Of the studied variants rs3743674 of the CDH1 gene (crude P=0.014 and adjusted p=0.000) evident significant association with breast cancer in Pakistani Pashtun population. Whereas TP53rs1042522 (crude P=0.251 and adjusted P=0.851) and ATM rs659243 (crude p=0.256 and adjusted p=0.975) showed no or negative association with breast cancer in study population.

**Conclusion:**

The present study demonstrates that CDH1rs3743674 polymorphism is associated with elevated breast cancer risk in the Pashtun ethic population of Khyber Pakhtunkhwa.

## Background

Breast Cancer (BC) is the most frequent type of all cancers reported worldwide. In males it accounts for less than 1% of the total cases reported 1 and less than 0.2% deaths are attributed to male from BC 2. However, BC in the females is more prevalent (32% of all cancers); and as whole it is the most common type of cancer reported with 1.67 million new expected cases 3, 4. Stated otherwise, 1 in every 4 of all new cases detected in females is BC and its incidence was increased by 20% between 2008 and 2012 throughout the world 5. Its incidence in Pakistani women is 2.5 times more than in other Asian countries such as India and Iran 6, and is the second main cause of death in such patients (American cancer society report 2013), with the proportion increasing further in post menopausal women after the age of 45 years 7. Moreover, the occurrence and death rate are increasing rapidly in developing countries 4.

Maximum cases arise infrequently in individuals with slight or no family antiquity of the disease 8 and only 5–10% is thought to be of genetic origin, although the core genetic cause is not yet recognized; still most of these cases are the results of alterations in the Breast cancer type 1 & 2 (BRCA1, BRCA2), Phosphatase and tensin homolog (PTEN), Cadherin-1 (CDH1), tumor protein p53 (TP53) or Serine/threonine kinase 11 (STK11) genes due to their association with a distinctive genetic cancer syndrome. In addition, Ataxia Telangiectasia Mutated gene (ATM), Checkpoint kinase 2 (CHEK2), BRCA1 Interacting Protein 1(BRIP1) and Recombinase (RAD51) genes association has been reported in certain cases^9^. Similarly, other less penetrant but more common genes such as ATM may describe the rest of genetic susceptibility to BC 10, 11. On the other hand, around 15–20% of such patients have a known domestic history with two or more first- and/or second-degree relatives having this disease. Thus a combination of genes along with environmental and lifestyles factors may contribute towards the development of disease^12^–^14^. Soaring affiliations have been shown by epidemiological studies of BC with risk factors such as family history of cancer, oral contraceptives, high exposure to estrogen, diet, ecological factors, premature puberty, and socioeconomic status of the patients 15–19.

Emerging evidences suggest that BC can arise due to changes in human cadherin-1 (E-cadherin/ CDH1) gene and tumor suppressor gene (TP53) 20–23. In case of any mutation in TP53 gene, loss of normal functions along with the abilities to produce tumorigenesis may develop^24^. Expression of E-cadherin either blackout or down regulation interferes with the veracity of intercellular adhesion junctions 25, 26, leading to decrease intercellular adhesion and increase cell motility that may permit cancerous cells to cross the base membrane and diffuse in the neighboring tissues 27. Polymorphic variants have been reported in CDH1 gene in a number of populations 28, 29. E-cadherin supporter consisting a C→A polymorphism associates a decreased in efficiency of the gene transcription 30, 31. Cattaneo et al 32 stated that the transcription factor binding capacity of A allele has a 68 % less compared to the C allele. Its existence shows more vulnerability to breast, colorectal, endometrial, prostate, lung and gastric cancers in various racial groups 30, 32,33.

In this study, the correlation was determined between *CDH1* rs3743674, *ATM* rs659243 and *TP53* rs1042522 polymorphisms of breast cancer risk in females of Khyber Pakhtoon Khawa, Pakistan. A large number of studies have been conducted on breast cancer patients globally, but no study on the subject has still been performed in this population. So we suppose to get a good perceptive of risk linked with such polymorphisms in the females of KP, Pakistan.

## Materials and Methods

### Sample collection & study population

A Case-Control study was designed consisting of 100 Familial Breast Cancer patients and 100 gender- and age-matched healthy volunteers, recruited from various Tertiary care hospitals including Institute of Radiotherapy & Nuclear Medicine (IRNUM) hospital, Peshawar, Khyber Teaching Hospital (KTH), Peshawar, and Hayatabad Medical Complex (HMC), Peshawar. Patients were histopathlologically confirmed with breast cancer. Case as well as control samples of whole blood were collected after through physical examination, informed written consent from patients or guardians authorizing the use of blood samples and their clinical data, in a properly labeled EDTA tubes. A detailed and carefully designed patient history proforma having information regarding breastfeeding duration, menarche and the menstrual cycle, use of oral contraceptives, age and demographic characteristics with lifestyle factor data were noted from the medical reports accompanied by a personal interviewer-administered questionnaire that was conducted by nurses in the presence of physicians 15. The study was approved by the ethical committee of the department of pharmacy, University of Peshawar via Ref. # 920/PHAR, dated 30^th^ October, 2018.

### Inclusion/Exclusion criteria

Inclusion and exclusion criteria of the patients and healthy individuals were as follow: (i) patients who have histopathologically confirmed BC with at least one first and/or second-degree relatives and (ii) aged between 25 to 65 years were included, while (i) patients who have no family history of BC or (ii) aged below 25 and above 65 years were excluded from the study.

Criteria for control subject's selection: (i) Normal healthy age-matched subjects of similar ethnicity and (ii) aged between 25 & 65 years and free from breast cancer.

The genomic study was conducted at the Genomic Center of Rehman Medical Institute, Hayatabad Peshawar, Pakistan, using Next Generation Sequencing (NGS).

### DNA Extraction

DNA was extracted using standard DNA extraction Kit (NovelGenomic DNA Mini Kit; cat. No. NG-S250), as per manufacturer's instructions. Quality of DNA was confirmed by running on 1% agarose gel and the quantity was checked by Qubit® fluorometer with the aid of dsDNA high sensitivity kit (Qubit, Cat. No. Q32851). After quantification of DNA, each sample was properly labeled, recorded according to the DNA concentration present and stored at –20°C for further analysis 21.

### DNA Quantification

Prior to Library Preparation, quantification of DNA was done using Qubit Fluorometer with the help of dsDNA high sensitivity kit (Qubit, Cat. #. Q32851) and the concentration was adjusted to 10 ng/µL.

### DNA pooling

DNA fractions extracted from of all samples (included in the study) were pooled according to the previously described protocols 34, 35. The process was carried out to simplify the sequencing process and to save the cost and time of analysis. Pooling was done by mixing an equimolar amount of DNA (100ng) from each individual sample and then subjected to further steps for libraries preparation and sequencing.

### Library Preparation

Illumina Nextera XT DNA library kit (Cat. No. FC-142-1123) was used to generate paired-end libraries (2101 bp) by properly following the manufacturer guidelines 36. Initial fragmentation of genomic DNA by Transposome into randomly sized DNA fragments were followed by a cleanup approach to remove transposomes adhering to DNA fragments to minimize interference in the subsequent steps, and DNA amplification using 12 cycles of thermal PCR.

Paramagnetic beads were used to eliminate fragments of less than 150–200 bp (unamplified) after the PCR amplification was completed. The exome amplified pieces of DNA (pre-selected genomic regions of interest) were then maintained, while non-specified DNA fragments were removed using biotinylated probes, as per the capture method plan. Using an Agilent 2100 Bioanalyzer (Agilent 228 Technologies), libraries were measured to confirm final DNA concentration.

Finally, the sequencing of the generated libraries was completed using the Illumina MiSeq NGS Machine. The sequence data generated by the Illumina MiSeq NGS Machine was saved in FASTQ format.

The study was conducted in the Center for Genomics, Rehman Medical Institute Hayatabad, Peshawar Pakistan.

### Bioinformatics Data Analysis

The FAST Q files obtained were subjected to different downstream analysis. These files were filtered on the basis of quality score using ‘Trimmomatic’ software to eliminate the Q30 & Q20 files and analyze Q40 & Q30 files only. For the data analysis and alignment, the newly identified sequence reads were aligned to a reference genome with the help of bioinformatics Burrows-Wheeler Aligner (BWA) software and BAM files were visualized on Integrated Genome Viewer (IGV). After alignment, differences between the reference genome, variant calling (vcf), and the newly sequenced DNA reads, were identified and analyzed.

### Statistical analysis

A chi-square test (χ2) was performed to know the relationship of genotypes of patients and healthy controls with breast cancer risk. Assessment of odd ratio (OR) and 95 % confidence intervals (CIs) was estimated through binary logistic regression. The P<0.05 was considered statistically significant. ORs were also calculated for various clinicopathological characteristics for both the mutation carriers and non-carriers. All of the statistical analysis was determined by applying the SPSS software, version 21.00.

## Results

### Basic demographic data of patients

Demographic as well as other characteristics such as use of oral contraceptives, duration of breast feeding, area of residence, education and history of breast cancer in the first and/or second degree relatives, were studied as given in the table # 1 & 2. Mean age of patients and control were 43.30 +18.41 and 46.96+18.41 respectively.

**Table 1:** Demographics detail of cases and controls

Variables	Case	Control	P value
**Age groups (yrs)**			0.085
15–30	14 (8.0%)	4 (2.3%)	
31–45	31 (17.6%)	32 (18.2%)	
46–60	37 (21.0%)	43 (24.4%)	
>60	6 (3.4%)	9 (5.1%)	
**Marital status**			0.136
Single	6 (3.4%)	12 (6.8%)	
Married	82 (46.6%)	76 (43.2%)	
**Drugs/contraceptive**			0.003
No Drugs	41 (23.3%)	61 (34.7%)	
Contraceptives	33 (18.8%)	14 (8.0%)	
Other Drugs	14 (8.0%)	13 (7.4%)	
**Socioeconomic status**			0.000
Poor	36 (20.5%)	33 (18.8%)	
Satisfactory			
Good, Well Off	50 (28.4%)	31 (17.6%)	
	2 (1.1%)	24 (13.6%)	

**Table 2:** Clinical Characteristics of patients and controls.

Variables	Groups	Case	Control	P Value
Menarche	12–13	53 (30.1%)	63 (35.8%)	0.51
	14–15	33 (18.8%)	21 (11.9%)	
	16–18	2 (1.1%)	4 (2.3%)	
Age at Menopause	N/A	45 (25.6%)	34 (19.3%)	0.18
	41–50	35 (19.9%)	34 (19.3%)	
	51–60	7 (4.0%)	20 (11.4%)	
	61+	1 (0.6%)	0 (0.0%)	
Age at First Pregnancy	No Pregnancy	3 (1.7%)	7 (4.0%)	0.42
	14–19	47 (26.7%)	46 (26.1%)	
	20–25	28 (15.9%)	28 (15.9%)	
	26–31	10 (5.7%)	7 (4.0%)	
No of Children	No Child	7 (4.0%)	9 (5.1%)	0.663
	1–3	20 (11.4%)	23 (13.1%)	
	4–6	39 (22.2%)	30 (17.0%)	
	7–9	16 (9.1%)	21 (11.9%)	
	10–12	6 (3.4%)	5 (2.8%)	
Duration of Breast Feeding	Below 1 Yr	10 (5.7%)	15 (8.5%)	0.233
	1–2 Yr	55 (31.3%)	44 (25.0%)	
	2–3 Yr	23 (13.1%)	29 (16.5%)	
Enlargement of Breast	No Increase	23 (13.1%)	69 (39.2%)	0.000
	Gradual Increase	53 (30.1%)	18 (10.2%)	
	Rapid Increase	12 (6.8%)	0 (0.0%)	
Breast Ulceration	Yes	11 (6.3%)	0 (0.0%)	0.320
	No	77 (43.8%)	81 (46.0%)	
Cyclical Pain	No Pain	33 (18.8%)	69 (39.2%)	0.000
	Cyclical Pain	24 (13.6%)	13 (7.4%)	
	Acyclical Pain	21 (11.9%)	3 (1.7%)	
	Other Pain	10 (5.7%)	3 (1.7%)	
Mobility	Non Mobile	36 (20.5%)	3 (1.7%)	0.000
	Mobile	52 (29.5%)	85 (48.3%)	
Tenderness	No	50 (28.4%)	86 (48.9%)	0.000
	On Self Palpation	28 (15.9%)	0 (0.0%)	
	On Deep Palpation	10 (5.7%)	1 (0.6%)	
Peaudorange	No/Normal	57 (32.4%)	88 (50.0%)	0.000
	Present	31 (17.6%)	0 (0.0%)	
Nipple Discharge	No Discharge	69 (39.2%)	88 (50.0%)	0.000
	Discharge	19 (10.8%)	0 (0.0%)	
Lymphedema	No Edema	67 (38.1%)	88 (50.0%)	0.000
	Edema	21 (11.9%)	0 (0.0%)	
Weight Loss	No Loss	68 (38.6%)	88 (50.0%)	0.000
	Weight Loss	20 (11.4%)	0 (0.0%)	
Liver Changes	No Change	60 (34.1%)	88 (50.0%)	0.000
	Degenerative Changes	19 (10.8%)	0 (0.0%)	
	Fatty/Enlarged	9 (5.1%)	0 (0.0%)	

Age groups and marital status showed no significant deviation (P>0.05) between patients and controls, whereas significant deviation (P>0.05) was found between users and non-user of drugs/contraceptives (P=0.003) and as well as in socioeconomic status groups.

According to different age group, majority of the patients 37 (21%) belongs to age group 46–60 years followed by 31(17.6%) [31–45years], 14(8%) [15–30years] (t test p value = 0.085).

All of the subjects were females and no male patient throughout the study was observed. Regarding regional distribution of the patients shown in table 1, the majority 53 (30.1%) of the patients belongs to Peshawar division. Patients of other regions like Mardan, Malakand, Kohat and Bannu divisions were also incorporated in the study. Majority of the patients 82 (93.3%) were found married (t test p value = 0.136). According to socio-economic status of the patients, majority of the patients were found in poor category, followed by satisfactory and then well off (t-test p value = 0.000). Highest incidence (30.1%) of BC was observed in Peshawar division, followed by Mardan (6.8%), Malakand, Kohat (2.8% each) and tribal districts (3.4%).

Different clinicopathological parameters like menarche, age at clinical stages (menopause, first pregnancy), no. of children, duration of breast feeding, enlargement of breast and ulceration, mobility, tenderness of breast, nipple discharge, Peaudorange, cyclical pains, Lymphedema, weight loss and liver changes were also studied (Table. 2). Significant deviation (P>0.05) between patients and controls was observed in terms of breast enlargement, cyclical pain, peaudorange, mobility, tenderness, nipple discharge, Lymphedema, weight loss and liver changes, whereas rest of the factors showed no significant deviation (P>0.05).

### Risk Variants reported in the study population

Whole Exome Sequencing (WES) results are shown in table 3. The WES reported three missense variants in the selected genes namely TP53rs1042522, CDH1rs3743674 and ATM rs659243 in the study population. WES reported risk variants were validated by using polymerase chain reaction restriction fragment length polymorphism.

**Table 3:** Whole Exome Sequencing (WES) results

	Gene	Variant	Homo%	Hetero%	HGVSc	HGVSp	Db SNP ID
**Cases**	TP53	G>G/C	26	78	NM _ 000546.5:c.215C>G	NP _ 000537.3:p. Arg 72 Pro	rs1042522
**Control**	TP53	G>G/C	66	34	NM _ 000546.5:c.215C>G	NP_000537.3:p. Arg 72 Pro	rs1042522
**Cases**	CDH1	C>C/T	70	30	NM_004360.3:c.48+6C>T	N/A	rs3743674
**Control**	CDH1	C>C/T	75	25	NM_004360.3:c.48+6C>T	N/A	rs3743674
**Cases**	ATM, C11ORF65	A>G/G	55	45	NM _ 000051.3:c.5948A>G	NP _000042.3:p.Asn1983Ser	rs659243
**Control**	ATM, C11ORF65	A>G/G	52	48	NM _ 000051.3:c.5948A>G	NP _000042.3:p.Asn1983Ser	rs659243

The allelic and genotypic frequencies of *TP53*rs1042522, *CDH1*rs3743674 and *ATM* rs659243 polymorphism of both controls and cases are given in Table 4. In case of CDH1rs3743674 risk allele was significantly high in Breast cancer patients compared to healthy controls. *CDH1*rs3743674 polymorphism (crude P=0.014 and adjusted P =0.000) was evident as risk variant and increases risk for breast cancer in the Pashtun ethnic population of Pakistan. *TP53*rs1042522 and *ATM* rs659243 polymorphism showed No/negative association with breast cancer in the present studied population.

**Table 4:** Genotypic, Allelic and Carriage Rate Frequencies of Tp53 CDH1Genes Polymorphism in Controls (n=88) and Familial Breast Cancer Cases (n=88)

Gene	Genotype /Allele	Case N (%)	Control N (%)	OR ( 95%CI )	P-Value	Adjusted OR ( 95%CI )	P-Value
TP53Variant	G>C/C	34(19.3%)	29(16.5%)	0.647 (0.308–1.361)	0.251	0.81 (0.1–6.89)	0.851
	G>G/C	32(18.2%)	30(17.0%)				
	Mutation not reported	22(12.5%)	29(16.5%)	Reference		Reference	--
CDH1.Variant	C>C/T	28(15.9%)	14(8.0%)	0.250 (0.082–0.759)	**0.014**	1.07 (0.05–22.59)	**0.000**
	C>T/T	63(34.1%)	40(29.1%)				
	Mutation not reported	07(4.0%)	14(8.0%)	Reference		Reference	--
ATM	A>G/C	55(31%)	52(29%)	0.560 (0.212–1.512)	0.256	1.07 (0.05–1.059)	0.975
	A>G/G	53(30%)	58(35%)				
	Mutation not reported	0(0.0)	0(0.0)	Reference	0	--	--

## Discussion

Genetic modification due to single nucleotide polymorphism in the genomic DNA has an involvement in cancer development 37–39. Polymorphism in the tumor suppressor *TP 53* and cell-cell adhesion *CDH1* genes is found to increase susceptibility for breast cancer 23,40. In Present study, three variants (*TP53rs1042522, CDH1rs3743674* and *ATM rs659243*) were screened for its association with BC in Pashtun ethnic population. Association of TP53 rs1042522 with breast cancer was reported 23. Previously Sekar et al described that over-expression of TP53 was considerably connected with the breast cancer development^41^. E-cadherin loss results in the dedifferentiation and incursion of the breast carcinoma 42. The current study concentrates on fascinating results including *CDH1* and *TP 53* and *ATM* genes mutations in patients, by comparison, to ethnically matched controls.

*TP53* emerges as an essential regulatory protein that acts as a multifunctional transcription factor to control the cell-cycle progression, restore DNA damage to keep the integrity of genome and induces apoptosis where stressors create abnormal and irreversible injury to eliminate the smashed cells 43–45. As the magnitude of cellular stress is linked with post-translational modifications of tumor suppressor gene, TP53 can be considered as a possible molecular signature for the study of breast cancer populations at high-risk 46–48. Literature shows the involvement of heterozygous Arg/Pro variant enhances the risk of breast cancer in the population. From the study it was observed that Arg/Pro incidence was 77.7 % in patients and 33.3 % in controls which shows a major association of this polymorphism with breast cancer. In Japani women, this polymorphism was reported with 48.9% in controls and with 50.0 % of patients for Arg/Pro heterozygosity^49^. In New York, the ratio of same polymorphism was 42.2 % and 35.4 % in patients and controls respectively. However, increase in breast cancer risk due to heterozygous genotype by 32 % was observed in the same population^50^. Lum et al. 51 reported a high prevalence of Arg/Pro heterozygosity with 47.5 %f controls and 51.0 % of patients in the Chinese population. All these results are in line with our findings.

In the current study, we found an alliance among the rs3743674 mutant and breast tumor (crude P= 0.014 and adjusted P=0.000). A high risk of breast cancer for Pro/Pro homozygosity (Odds Ratio = 2.38; P = 0.046) was also found in Austrian^52^ and Japani population (Odds Ratio = 2.14; 95 % confidence interval=1.21–3.79) 49. It has an important role in the risk of hormone receptor (ER- positive) breast cancer with adjusted OR = 2.04, P = 0.04 in Japanese women 53. A large quantity of genotype carrying the pro allele (Pro/Pro and Pro/Arg) (OR = 1.47, P = 0.014; 95 % CI = 1.08 – 2.00) and its frequency was found in the Swedish population 54. Lum and his colleagues established a biologically relation to the presence of homozygosity for proline with breast cancer patients in Chinese women, with 16.3% for controls and 22.1% for patients 51.

All these results are in line with our findings. Moreover our findings are not in line with those studies which establish a high frequency of homozygosity of Arg in breast cancer patients as reported in Turkish 55, 56, Arab 48, Iranian 57, Greece 58, and Southern Brazilian populations^59^. Keshava et al found high commonness of Arg allele in Caucasian breast cancer patients 60, and higher prevalence of the Arg allele was found by Ohayon et al.^61^ in the Ashkenazi and non-Ashkenazi Jews.

Reports have shown that TP 53 Arg 72 variant is more efficient in the initiation of apoptosis 62–64. Therefore the Pro allele is considered to be mostly responsible for reduction in apoptosis leading to breast cancer.

E-cadherin has an important role in cellular differentiation, inter-cellular adhesion and cell signaling. Studies have shown the association of different types of cancers with CDH1 rs3743674 polymorphism, however some other reports have shown no significant relation. The non-significant relation may be due to genetic and ethnic variability of the patients and controls that might be responsible for this inequality among these information. Our findings are in line with the majority of the studies conducted in different populations. The genetic changes in CDH1 gene along with cellular polarity and cell-to-cell adhesion, is finally accountable for the metastasis and tumor progression 26, 28, 65.

A previous study relates *CDH1*rs3743674 and *TP53*rs1042522 polymorphisms with the risk and breast cancer progression 53, 54, 58, 66, 67. In the present study we have screened the presence of the polymorphism in TP53 codon 72 and *CDH1* rs3743674 and *ATM* rs659243 genes in breast cancer for the first time in patients of Khyber Pakhtunkhwa population to evident the association of the aforementioned gene varints with BC in study population.

## Conclusion

Our results show a significant association of CDH1 rs3743674 polymorphism with increasing risk of breast cancer in the Pashtun population of Pakistan. This study will help to provide a plate form for future diagnosis and treatment of breast cancer patients. Similar projects should be designed by national governmental agencies to screen and pinpoint genetically susceptible individuals and awareness campaigns are needed regarding genetic susceptibility and environmental risk factors be initiated in general public.
